# Outcomes with esketamine nasal spray in patients with or without earlier partial or full response: post hoc analyses of ESCAPE TRD

**DOI:** 10.1192/j.eurpsy.2025.861

**Published:** 2025-08-26

**Authors:** A. H. Young, Y. Godinov, B. Rive, T. Ito, J. Buyze, A. J. Oliveira-Maia, A. Fagiolini, N. Cardoner, P. Gorwood, R. S. McIntyre, K. Demyttenaere

**Affiliations:** 1 Department of Psychological Medicine, Institute of Psychiatry, Psychology and Neuroscience, King’s College London, London; 2 South London and Maudsley NHS Foundation Trust, Bethlem Royal Hospital, Beckenham, London, United Kingdom; 3 Janssen EMEA, Sofia, Bulgaria; 4 Janssen EMEA, Paris, France; 5 Janssen EMEA, High Wycombe, United Kingdom; 6 Janssen Pharmaceutica NV, Beerse, Belgium; 7 Champalimaud Research and Clinical Centre, Champalimaud Foundation; 8 Universidade NOVA de Lisboa, Lisbon, Portugal; 9 Department of Molecular Medicine, University of Siena School of Medicine, Siena, Italy; 10 Institut d’Investigació Biomèdica Sant Pau, Hospital de la Santa Creu i Sant Pau, Barcelona; 11 CIBERSAM, Carlos III Health Institute, Madrid; 12 Universitat Autònoma de Barcelona, Barcelona, Spain; 13 Université Paris Cité, Institute of Psychiatry and Neuroscience of Paris (IPNP), INSERM U1266; 14 GHU-Paris Psychiatrie et Neurosciences, Hôpital Sainte Anne, Paris, France; 15 University of Toronto; 16 Braxia Scientific, Toronto, Canada; 17 Universitair Psychiatrisch Centrum KU Leuven, Leuven, Belgium

## Abstract

**Introduction:**

There are limited data to guide treatment continuation decisions for clinicians caring for patients with treatment resistant depression (TRD). Identifying the magnitude of early improvement (at Weeks 4 and 8) as a predictor of long-term outcomes for TRD can guide treatment continuation decisions.

**Objectives:**

To evaluate the probability of achieving response or remission by Week 32 in patients with TRD after 4 or 8 weeks of esketamine nasal spray (ESK-NS) treatment, flexibly dosed in combination with an ongoing selective serotonin/serotonin-norepinephrine reuptake inhibitor (SSRI/SNRI).

**Methods:**

ESCAPE‑TRD was a randomised phase IIIb trial comparing the efficacy of ESK-NS versus quetiapine extended release, both in combination with an ongoing SSRI/SNRI, in patients with TRD (Reif *et al.* NEJM 2023; 389 1298–309). Remission was defined as a Montgomery-Åsberg Depression Rating Scale (MADRS) total score ≤10, and partial response and response as ≥25% and ≥50% improvements, respectively, in total MADRS score (or remission). Long-term outcomes were based on the best outcome on-treatment across 32 weeks (≥1 instance of response or remission) from earliest outcome endpoint onwards. Non-responder imputation (NRI) was applied after treatment discontinuations.

**Results:**

336 patients were randomised to ESK-NS; 334 received ≥1 dose. **Table 1** shows long-term outcomes following at least partial response, response or no partial response at Weeks 4 and 8. For example, among those who had at least a partial response at Week 4, 94.1% and 79.8% had response and remission by Week 32, respectively.

**Image 1:**

**Figure:**
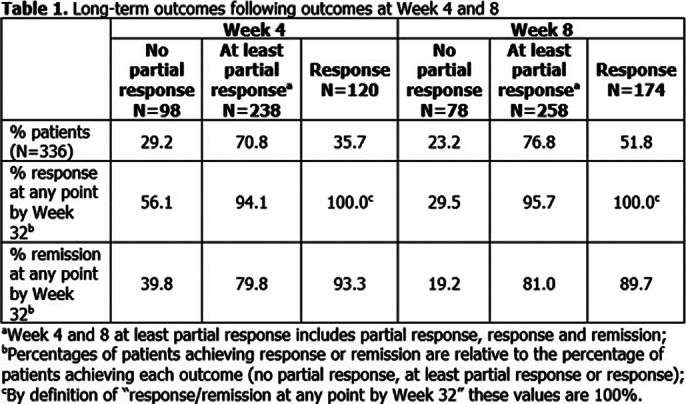

**Conclusions:**

This analysis demonstrated a relationship between short- and long-term outcomes. Presence of at least partial response at Week 4 led to more favourable outcomes by Week 32. Moreover, most patients with response by Week 4 achieved remission by Week 32. Continued symptom improvements were observed beyond the induction phase even in some patients with no partial response at Week 4.

**Disclosure of Interest:**

None Declared

